# ESPO AND BUTLER-WILLIAMS SCIENTIFIC SYMPOSIUM: CAREER DEVELOPMENT TO PROMOTE DIVERSITY, DISCOVERY, AND AGING

**DOI:** 10.1093/geroni/igad104.0383

**Published:** 2023-12-21

**Authors:** Jamaine Davis, Brianna Morgan, Patricia Jones

## Abstract

The NIA’s Butler-Williams Scholars Program and GSA’s ESPO Section are united in providing career development opportunities in a manner that promotes leadership, diversity, and inclusivity. This year’s theme challenges our emerging scholars to embrace diversity and discovery while thinking—or rethinking—about the perspectives of older adults. Disparities in health associated with race/ethnicity, lived experience, sociocultural and socioeconomic factors, as well as access to and provision of health care are chief concerns of our aging population. GSA’s early career professionals and 2022 alumni of the prestigious NIA Butler-Williams Scholars Program address these issues. Dr. Shekinah Fashaw-Walters will discuss her work examining how high-quality providers influence high quality of healthcare, particularly for Black patients. Dr. Sara LaHue will discuss how biological age can be a measure associated with delirium or neuronal inury markers in a cohort of hip fracture patients. Dr. Imilce Rodrigues-Fernandez will present on the importance of identifying pathways that can lead to improvements in stem cell function that can improve age-related tissue degeneration. how both demographics and genetic factors contribute to cognitive performance. The final speaker, Dr. Helena Zomer will present findings on the mechanism by which a hormone can extend healthspan and longevity. The featured talks by rising stars deepen our understanding of the influence of diversity and key discoveries so we can reimagine aging research.

